# Computed Tomographic Measurements of Thigh Muscle Cross-Sectional Area and Attenuation Coefficient Predict Hip Fracture: The Health, Aging, and Body Composition Study

**DOI:** 10.1359/jbmr.090807

**Published:** 2009-08-03

**Authors:** Thomas Lang, Jane A Cauley, Frances Tylavsky, Douglas Bauer, Steven Cummings, Tamara B Harris

**Affiliations:** 1 Department of Radiology, University of CaliforniaSan Francisco, CA, USA; 2 Department of Epidemiology, University of PittsburghPittsburgh, PA, USA; 3 University of Tennessee Health Science CenterMemphis, TN, USA; 4 Department of General Internal Medicine, University of CaliforniaSan Francisco, CA, USA; 5 San Francisco Coordinating Center, California Pacific Medical CenterSan Francisco, CA, USA; 6 Laboratory of Epidemiology, Demography, and Biometry, National Institute on Aging, National Institutes of HealthBethesda, MD, USA

**Keywords:** Hip fracture, Sarcopenia, Osteoporosis, Fatty infiltration of skeletal muscle, Computed Tomography, Aging

## Abstract

Fatty infiltration of muscle, myosteatosis, increases with age and results in reduced muscle strength and function and increased fall risk. However, it is unknown if increased fatty infiltration of muscle predisposes to hip fracture. We measured the mean Hounsfield unit (HU) of the lean tissue within the midthigh muscle bundle (thigh muscle HU, an indicator of intramuscular fat), its cross-sectional area (CSA, a measure of muscle mass) by computed tomography (CT), bone mineral density (BMD) of the hip and total-body percent fat by dual X-ray absorptiometry (DXA), isokinetic leg extensor strength, and the Short Physical Performance Battery (SPPB) in 2941 white and black women and men aged 70 to 79 years. Sixty-three hip fractures were validated during 6.6 years of follow-up. Proportional hazards regression analysis was used to assess the relative risk (RR) of hip fracture across variations in thigh muscle attenuation, CSA, muscle strength, and physical function for hip fracture. In models adjusted by age, race, gender, body mass index, and percentage fat, decreased thigh muscle HU resulted in increased risk of hip fracture [RR/SD = 1.58; 95% confidence interval (CI) 1.10–1.99], an association that continued to be significant after further adjustment for BMD. In models additionally adjusted by CSA, muscle strength, and SPPB score, decreased thigh muscle HU but none of the other muscle parameters continued to be associated with an increased risk of hip fracture (RR/SD = 1.42; 95% CI 1.03–1.97). Decreased thigh muscle HU, a measure of fatty infiltration of muscle, is associated with increased risk of hip fracture and appears to account for the association between reduced muscle strength, physical performance, and muscle mass and risk of hip fracture. This characteristic captures a physical characteristic of muscle tissue that may have importance in hip fracture etiology. © 2010 American Society for Bone and Mineral Research

## Introduction

Increases in the adiposity of skeletal muscle and other tissues have been linked at the cellular level to underlying age-related processes such as redox damage, which can result in mitochondrial dysfunction and impaired oxidative metabolism. These changes are associated with numerous deleterious health conditions in the growing elderly population, including metabolic conditions such as insulin resistance and diabetes.(1–3) Aging has been linked to the increasing tendency of precursor cells such as bone marrow mesenchymal cells or muscle satellite cells to express an adipocytic instead osteoblastic or myocytic phenotype with age. Moreover, in skeletal muscle, age-related decreases in the ability of muscle fibers to process trigylceride results in increased storage of lipid in the form of droplets that form along the cell membrane.

The infiltration of skeletal muscle by noncontractile components such as lipid, along with loss of muscle mass, appears to contribute to age-related losses in skeletal muscle function. This results in loss of muscle strength and reduced lower extremity performance, both of which confer increased risk of outcomes such as loss of mobility, falls, and skeletal fractures. In addition, impaired muscle strength and reduced physical function may in themselves cause loss of bone strength owing to lower skeletal loading from reduced weight bearing and muscle loading. Further, fatty infiltration into muscle is also associated with metabolic disorders that may increase risk of falling owing to impaired vision and/or limb pain.(4,5) Bone loss owing to reduced loading of the skeletal also may be exacerbated by the loss of central fat depots, which also may have negative skeletal effects through reduced production of estrogen in the adipose tissues.

Although epidemiologic studies have correlated measurements of muscle and fat mass with fracture-related measures of bone mineral density (BMD), functional decline, and metabolic dysfunction in the elderly,(6–8) there is little information about the relationship of body-composition variables to incident hip fracture, the most serious consequence of osteoporosis. To address this issue, we analyzed data from the Health Aging and Body Composition (Health ABC) Study, a prospective cohort study of 3075 black and white men and women in Pittsburgh, Pennsylvania, and Memphis, Tennessee, designed to characterize changes in body composition as a pathway by which weight-related health conditions contribute to disease and disability in the elderly. To determine whether body composition measures predict hip fractures and to understand whether these measures act as surrogates for function-related hip fracture risk factors, we correlated measures of computed tomographic (CT) and dual X-ray absorptiometric (DXA) body composition, bone density, and physical function obtained at baseline with incident hip fracture in the cohort.

## Materials and Methods

### Study population

The Health, Aging, and Body Composition (Health ABC) Study cohort includes 3075 black and white men and women, and its methods have been described in detail.(9–11) White participants were recruited from a random sample of Medicare beneficiaries residing in ZIP codes from the metropolitan areas surrounding Pittsburgh, Pennsylvania, and Memphis, Tennessee. All black subjects residing in these areas were considered to be potential participants and were solicited for participation. Eligibility criteria included age 70 to 79 years with self-report of no difficulty walking one-quarter mile or climbing 10 steps without resting; no difficulty performing basic activities of daily living; and no reported use of a cane, walker, crutches, or other special equipment to get around. Of the 3075 participants, we excluded those with missing data on incident mobility limitations (*n* = 7), muscle area or muscle attenuation (*n* = 53), total-body fat mass (*n* = 14), or muscle strength (*n* = 370; see “Muscle Strength” below). A total of 2631 participants (85.6% of original cohort, 1286 men and 1345 women) were available for analysis.

### Ascertainment of hip fractures

Participants were contacted every 6 months, including yearly clinic visits, and were asked about recent medical history, including fractures. All reported hip fractures were confirmed by radiographic report.

### Muscle mass

The cross-sectional area (CSA) of muscle in both thighs was used as a measure of muscle mass. Muscle area was measured by computed tomography (Memphis clinic site: Somatom Plus 4, Siemens, Erlangen, Germany, or PQ 2000S, Marconi Medical Systems, Cleveland, OH, USA; Pittsburgh clinic site: 9800 Advantage, General Electric, Milwaukee, WI, USA) as described previously.(1,2,12) In short, a single 10 mm thick axial image (120 kVp, 200 to 250 mA) of both thighs was obtained at the midfemur, and intermuscular and visible intramuscular adipose tissue was separated from subcutaneous adipose tissue by drawing contours along the deep fascial plane surrounding the thigh muscles. The total midthigh CSA of nonadipose, nonbone tissue within the deep fascial plane was used as a measure of muscle mass.

### Measurement of muscle attenuation coefficient from CT scan

**Fig. 1:**
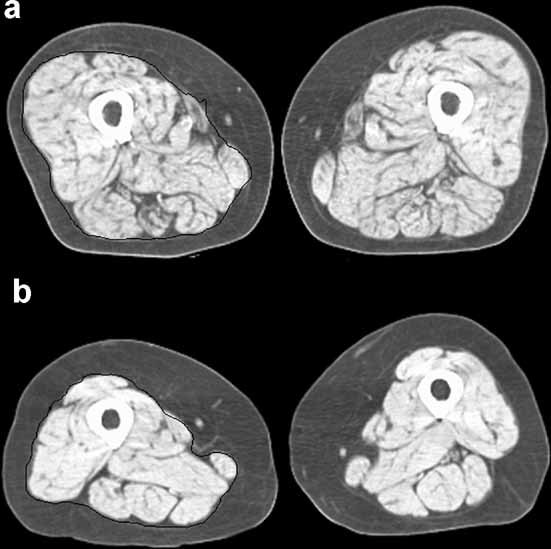
Representative CT images of the midthigh showing in black the outline of the region of interest encompassing the thigh muscle bundle used for area and attenuation measurements in this study. (a) Axial image showing extensive fatty infiltration of the muscle and having a thigh muscle lean tissue attenuation coefficient of 26 HU. (b) Axial image with a thigh muscle lean tissue attenuation coefficient of 38.6 HU.

The mean attenuation coefficent [measured in Hounsfield units (HU)] of thigh muscle tissue obtained by CT scan,(4,10,13) excluding intermuscular and intramuscular adipose tissue lying interior to the deep fascial plane surrounding the muscle, was used as an indicator of fat infiltration into the muscle. Lower thigh muscle Hounsfield units indicates greater fat infiltration.(4) A previous study showed that the interscan reproducibility of the thigh muscle Hounsfield unit measurement is 0.85%, a figure that includes the errors associated with repositioning the subject in the scanner and reanalyzing the image.(4) Figure 1 shows examples of midthigh scans in two subjects having differing levels of fatty infiltration.

### Bone mineral density (BMD)

Areal BMD (aBMD, g/cm^2^) of the total hip was assessed by DXA (Hologic QDR 4500A, Version 9.03, Bedford, MA, USA). Identical scan protocols were used for all participants.

### Muscle strength

The maximal isokinetic strength of the knee extensors (Nm) was assessed using a KinCom 125 AP Dynamometer (Chattanooga, TN, USA) at 60°/s and was calculated as the average of at least three and up to six reproducible and acceptable trials. Participants with a systolic blood pressure of 200 mm Hg, a diastolic blood pressure of 110 mm Hg, or who reported a history of cerebral aneurysm, cerebral bleeding, bilateral total knee replacement, or severe bilateral knee pain were excluded from testing (12.7% of original cohort).

### Lower extremity performance

A Short Physical Performance Battery (SPPB) was adapted from the lower extremity performance tests used in the Established Populations for the Epidemiologic Studies of the Elderly (EPESE).(14,15) The SPPB used in this study(16) consists of three tasks: time for five repeated chair stands performed without arms to push off, time able to hold a semitandem position up to 30 seconds, and time to complete a 6 m walk to determine usual gait speed. Each of these timed measures was scored from 0 to 4 and then summed for a total score of 0 to 12, with 12 being the most functional score.

### Covariates

Demographic covariates included age, race, gender, and clinical site. Anthropometric covariates included body mass index (BMI), standing height, and total body percentage of fat. Standing height was measured using a wall-mounted stadiometer and body weight with a standard balance scale. In order to adjust for the overall adiposity of subjects, total body percentage of fat was measured using DXA (Hologic QDR-4500, Bedford, MA, USA). Health-related covariates included a chronic disease index, physical activity in the week prior to the examination, and a self-rated health score. The chronic disease index(10) was assessed as the total number of 11 chronic health conditions identified using self-report and medications brought to the clinic and recorded as part of the examination. These conditions included cancer, myocardial infarction, congestive heart failure, depression, diabetes, hypertension, knee osteoarthritis, osteoporosis, peripheral arterial disease, pulmonary disease, and gastrointestinal disease. Physical activity was defined as the time spent on high- and moderate-intensity exercise and was represented as a total metabolic equivalent value (kcal/week). Self-rated health was categorized as excellent/very good, good, or fair/poor. Additional covariates included alcohol consumption (drinks/week) and smoking (total consumption in pack-years). Data also were adjusted for level of education and cognitive status assessed with the Teng Modified Mini-Mental Status Examination.(17)

### Statistical methods

Statistical analyses were carried out using the SAS statistical analysis program (SAS Institute, Cary, NC, USA). Mean values and standard deviations were computed for each race and gender subgroup for the predictor variables and covariates, and the distribution of each variable was examined to determine normality. To determine differences in key predictor measures and covariates between subjects who incurred hip fracture and nonfractured subjects, multivariate linear regression analysis (GLM procedure) was employed to compute least-squares means for the two groups adjusted for age, race, clinical site, and gender. Proportional hazards regression analyses (PHREG procedure) were employed to determine the individual associations of SPPB score, leg muscle strength, thigh muscle CSA, and fatty infiltration into muscle (thigh muscle HU values) with incident hip fracture. The analysis involved several models with varying adjustments. Models 1 and 2 included adjustments for age, gender, race, standing height, BMI, and percentage of body fat. Model 1 examined thigh muscle HU value, thigh muscle CSA, SPBB score, and leg muscle strength as individual predictors, whereas model 2 entered these variables simultaneously in the regression analysis. Model 3 added chronic disease index, physical activity, and self-rated health to the covariates of models 1 and 2. Model 4 included the covariates of model 3 but added cognitive status, drinking, smoking, educational level, and total hip BMD by DXA. To examine whether there was an expected linear relationship between quantile of thigh muscle HU value and incident hip fracture, the data were stratified into tertiles of thigh muscle HU value, and proportional hazards regression analysis was employed to estimate hazard ratios for hip fracture incidence for each tertile after adjustment for age, height, BMI, gender, race, clinical site, BMD, self-reported health, chronic disease index, and physical activity.

## Results

### Descriptive statistics and fracture/control differences

The characteristics of the study sample as a function of gender and race are shown in Table 1. Although whites made up roughly half the study population, they accounted for nearly 80% of the incident fractures. Table 2 compares least-squares mean values in subjects who incurred hip fractures with nonfractured subjects after adjustment for age, race, gender, and study site. Subjects who incurred fractures during the follow-up period were older, had a lower percentage of fat, lower muscle strength, lower SPPB score, lower self-rated health, and lower total hip BMD.

**Table 1:** Characteristics of the Health, Aging, and Body Composition Study Population

	White men (*N* = 857)	Black men (*N* = 513)	White women (*N* = 791)	Black women (*N* = 672)
Number of hip fractures	17	3	29	14
Pittsburgh Clinic (%)	50.3	51.1	46.2	53.6
Age (years)	73.9 (2.9)	73.4 (2.7)	73.6 (2.8)	73.4 (2.9)
Height (cm)	173.2 (6.3)	172.8 (6.8)	159.5 (5.9)	159.5 (6.4)
Total % fat	29.9 (4.7)	28.0 (5.2)	40.1 (5.4)	40.1 (5.9)
BMI (kg/m^2^)	27.0 (3.7)	27.1 (4.2)	26.0 (4.4)	29.5 (5.6)
Thigh muscle CSA (cm^2^)	127.2 (19.3)	138.6 (24.6)	85.2 (13.6)	101.3 (16.7)
Thigh muscle attenuation (HU)	37.5 (6.3)	37.0 (6.4)	34.8 (6.3)	32.6 (6.9)
Knee extensor strength (Nm)	131.2 (33.2)	135.8 (37.0)	78.6 (19.9)	86.0 (23.1)
SPPB score	10.6 (1.3)	9.9 (1.7)	10.0 (1.5)	9.5 (1.7)
Total hip BMD (g/cm^2^)	0.943 (0.143)	1.017 (0.157)	0.765 (0.126)	0.856 (0.150)
Chronic disease index	2.4 (1.5)	2.5 (1.5)	2.3 (1.4)	2.4 (1.4)
Physical activity (kcal/week)	84.8 (66.0)	83.0 (78.7)	84.3 (60.0)	81.3 (75.2)
Cognitive status	92.4 (6.1)	84.7 (10.1)	93.6 (5.4)	87.4 (8.8)
Smoking (pack-years)	27.8 (33.6)	23.1 (26.0)	12.7 (24.3)	11.9 (21.6)
Alcohol consumption (% more than 1 drink per week)	45.1	26.1	30.0	9.7
Education (% with low education)	14.5	48.6	10.5	39.2
Self-rated health (% excellent/very good)	53.7	34.7	50.9	34.3

**Table 2:** Comparisons of Key Measures for Subjects With and Without Hip Fracture After Adjustments for Age, Site, Race, and Gender

Variable	Fracture	Nonfracture
Age (years)^a^	75.7***	73.6
Site (% from Pittsburgh)^b^	47.2	49.1
Race (% white)^b^	73.0*	57.8
Gender (% female)^b^	68.2*	51.2
Height (cm)^c^	165.9	166.0
Total % fat^c^	33.6*	35.1
BMI (kg/m^2^)^c^	26.3	27.3
Thigh muscle CSA (cm^2^)^c^	108.1	111.5
Thigh muscle attenuation (HU)^c^	34.2	35.5
Knee extensor strength (Nm)^c^	98.5*	107.0
SPPB score^c^	9.6*	10.1
Total hip BMD (g/cm^2^)^c^	0.746**	0.889
Chronic disease index	2.7	2.4
Physical activity (kcal/week)^c^	70.8	83.8
Cognitive status	88.9	90.2
ng (packs-yr) (2)	22.9	18.7
Alcohol Consumption (% more than 1 drink per week) (3)	57.1	49.8
Education (% less than high school) (3)	17.7	25.6
Self-rated health (% excellent/very good) (3)	30*	45

Statistical significance levels: *p < .05; **p < .01; ***p < .001.

a Least-squares mean adjusted for site, race, and gender.

b Fisher's exact test.

c Least-squares mean adjusted for age, site, race, and gender.

### Associations of muscle and performance measures with hip fracture

**Fig. 2:**
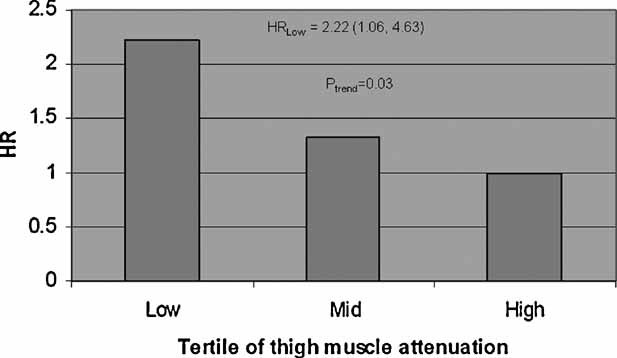
Plot of results of proportional hazards regression analyses. Hazard ratios (HRs) for incident hip fracture per tertile of thigh muscle attenuation after adjustment for age, height, BMI, gender, race, clinical site, self-reported health, chronic disease index, physical activity, and total femur BMD by DXA. HR values are normalized to the highest tertile, which is set to a value of 1.0. The trend of decrease in HR with increasing tertile is statistically significant. The lowest tertile of thigh muscle attenuation is significantly associated with incident hip fracture.

Table 3 shows individual associations of muscle strength, SPPB score, thigh muscle CSA, and thigh muscle HU value with incident hip fracture after adjustment for age, height, BMI, total percentage of fat, race, gender, and clinical site. Increased risk of hip fracture (model 1) was conferred by lower muscle strength [relative risk/standard deviation (RR/SD) = 1.83; 95% confidence interval (CI) 1.22–2.72], lower SPBB score (RR/SD = 1.21; 95% CI 1.06–1.39), smaller thigh muscle CSA (RR/SD = 1.65; 95% CI 1.16–2.34), and lower thigh muscle HU value (RR/SD = 1.58; 95% CI 1.18–2.12). After adding total femur BMD to the covariates of model 1, only thigh muscle HU value remained significantly associated with incident hip fracture (RR/SD = 1.46; 95% CI 1.08–1.97). When leg muscle strength, SPBB score, thigh muscle CSA, and thigh muscle muscle HU value were entered simultaneously in the proportional hazards model (model 2), only thigh muscle HU value was significantly associated with incident hip fracture (RR/SD = 1.41; 95% CI 1.01–1.93), an association that remained marginally statistically significant after adjustment for total femur BMD. Figure 2 shows hazard ratios per tertile of thigh muscle HU value. Only the lowest tertile of attenuation was significantly associated with an increased risk of hip fracture (RR/SD = 2.22; *p* = .03), but the trend for decreasing fracture risk with increasing tertile was statistically significant (*p* = .03).

**Table 3:** Proportional Hazard Regression Analyses Estimating Adjusted Hazard Ratios for Hip Fracture per SD Decrease (95% Confidence Intervals) of Muscle Strength, SPPB Score, Thigh Muscle Cross-Sectional Area, and Thigh Muscle Attenuation (a Measure of Fatty Infiltration)

	Model 1	Model 1 + BMD	Model 2	Model 2 + BMD
Knee extensor strength	1.83 (1.22–2.72)	1.32 (0.87–2.00)	1.49 (0.94–2.37)	1.31 (0.82–2.09)
SPPB score	1.21 (1.06–1.39)	1.08 (0.93–1.27)	1.13 (0.95–1.34)	1.03 (0.85–1.24)
Thigh muscle CSA	1.65 (1.16–2.34)	1.03 (0.71–1.50)	1.35 (0.89–2.03)	1.03 (0.66–1.61)
Thigh muscle attenuation	1.58 (1.18–2.12)	1.46 (1.08–1.97)	1.41 (1.01–1.93)	1.44 (1.02–1.99)

*Note:* Analysis results are shown for performance and muscle CT variables entered individually (model 1) and simultaneously (model 2) with and without adjustment for total femur BMD by DXA. Adjustment covariates: age, race, gender, clinic, height, BMI, and percentage of fat. Model 1: Performance and muscle CT variables entered individually with adjustment for covariates. Model 2: Performance and muscle CT variables entered simultaneously with adjustment for covariates.

### Associations of thigh HU value and total hip BMD with hip fracture after adjustment for anthropometric, demographic, and health-related covariates

Table 4 shows thigh muscle HU value as a predictor of hip fracture after adjustment for total femur BMD and different combinations of covariates. In model 3, thigh muscle HU value was independently associated with incident hip fracture (RR/SD = 1.51; 95% CI 1.13–2.03) after adjustment for age, race, gender, clinic, height, BMI, percentage of fat, self-reported health, chronic disease index, and physical activity. When total hip BMD was added to the model 3 covariates, the association between thigh muscle HU value and incident hip fracture remained statistically significant (RR/SD = 1.39; 95% CI 1.03–1.50), but the association became insignificant when a final adjustment for cognitive status, alcohol use, smoking, and education was carried out in model 4 (RR/SD = 1.35; 95% CI 0.99–1.83).

**Table 4:** Proportional Hazard Regression Analyses Estimating Adjusted Hazard Ratios for Hip Fracture per SD Decrease (95% Confidence Intervals) of Muscle Attenuation With Adjustment for Different Covariates

	Model 3	Model 3 + Total Femur BMD	Model 4
Thigh muscle attenuation	1.51 (1.13–2.03)	1.39 (1.03–1.50)	1.35 (0.99–1.83)
Total femur BMD	—	3.63 (2.68–4.92)	3.61 (2.66–4.91)

Covariates: Model 3: Age, race, gender, clinic, height, BMI, percentage of fat, self-reported health, chronic disease index and physical activity. Model 4: Model 3 + BMD and cognitive status, alcohol use, smoking, and education.

## Discussion

Aging entails a loss of muscle mass and infiltration of muscle tissue by lipid and other noncontractile components. Skeletal muscle fat exists in the form of extramyocellular lipid contained in adipocytes embedded between muscle fibers as well as intramyocellular lipid contained in droplets of triglyceride formed on muscle cell membranes. Loss of muscle mass and increased fatty infiltration are manifested in midthigh CT images as loss of muscle bundle CSA and decreased HU value of muscle tissue.

Previous reports describing our cohort have studied the association between thigh muscle HU value and measures of total body adiposity. Goodpaster and colleagues noted that increasing thigh muscle HU value was inversely correlated with BMI (*r* = –0.4), total body fat (*r* = –0.5), and total body percentage of fat (*r* = –0.5). On the other hand, thigh muscle CSA was positively associated with BMI (*r* = 0.6), indicating that individuals with more body fat have larger muscles but tend to have a smaller proportion of that fat in the muscle.(13)

Previous studies in our cohort have correlated measures of thigh muscle HU value and CSA with measures of muscle strength and lower extremity performance (SPBB score), which were correlated with incident hip fracture in the present study [hazard ratio (HR) = 1.83 and 1.21 for strength and SPBB score, respectively) and others.(18) Goodpaster and colleagues showed that increasing thigh muscle HU value was associated with higher values of thigh muscle specific torque, a measure of muscle quality obtained by normalizing the torque by the muscle CSA (*r* = 0.26; *p* < .0001).(13) Visser and colleagues examined the association of thigh muscle HU value with lower extremity performance (LEP, comparable with our SPBB score) in black and white men and women.(2) Within each race and gender group, they found that increasing thigh muscle HU value was associated with increasing LEP score, even after age, total body fat, education, physical activity, health status, and thigh muscle CSA were taken into account. Thus we hypothesized that thigh muscle HU value would be a predictor of incident hip fracture.

Our findings supported this hypothesis. Even after adjustments for age, gender, race, BMI, and total percentage of fat were taken into account, a decrease of 1 SD in thigh muscle HU value conferred a roughly 50% increase in hip fracture risk, a predictive value comparable with those conferred by reduced muscle mass (as reflected by thigh muscle CSA), muscle strength, and performance score. When health-related covariates including the chronic disease index and self-reported health were added to the model, the association of thigh muscle HU value with hip fracture was slightly reduced, supporting the idea that chronic diseases such as diabetes(19) and hypertension(20) also may contribute to the hip fracture association of the thigh muscle HU measurement. When thigh muscle HU value, thigh muscle CSA, muscle strength, and SPBB score were combined simultaneously in a prediction model, only the thigh muscle HU value continued to be weakly but independently associated with incident hip fracture, indicating that the thigh muscle HU value could be acting as a surrogate measure for the strength and performance battery measurements. These measurements obtained at the midthigh are consistent with an earlier cross-sectional study indicating an association with hip fracture of the cross-sectional area and HU values of hip extensor, abductor, adductor, and flexor muscle groups.(21) Because information on surrounding musculature is available from quantitative CT scanning of the hip, which is in limited clinical usage in osteoporosis assessment, these findings support further exploration of muscle size and density measurements as an adjunct to bone density for fracture risk assessment.

Fatty infiltration of muscle can affect hip fracture risk by modifying both muscle strength and bone strength. Muscle mass and quality are associated with skeletal density and geometry owing to mechanical loading forces on bone.(22–24) Moreover, fatty infiltration of bone and muscle tissue may be driven by the same underlying metabolic processes,(3) and individuals with high fat content in their muscles may tend to have weaker bones with high marrow adipose content. We addressed this question by examining the association of thigh muscle HU value with hip fracture in models adjusted and unadjusted by hip BMD. In these models, even when hip BMD was taken into account, a 1 SD decrease in thigh muscle HU value conferred a nearly 40% increase in the risk of hip fracture.

Our study has both strengths and weaknesses. The strengths of this novel study of hip fracture risk factors include the prospective study design and the combination of bone density, muscle imaging, and physical function measurements. However, this study also has several weaknesses. The number of fractures in our cohort was relatively small, resulting in large confidence intervals, particularly after adjustments for a large number of covariates. Although our cohort contained subjects of both genders and was multiracial, a relatively small number of hip fractures was contributed by black participants or by men, and most of the data came from white men and women. Finally, our study addressed the association of thigh muscle CSA and HU value across a relatively narrow age range, and the associations reported in this study may not apply to younger or older men and women. Thus further studies will be required to establish race- and gender-specific differences in the relationship between muscle size and composition variables and incident hip fracture.

In conclusion, we observed that thigh muscle HU value, an image-derived physical measurement capturing the age-related infiltration of muscle tissue by fat, predicts hip fracture independently of BMD and appears to act as a surrogate for the roles of muscle strength and physical function as hip fracture risk factors. Thus measurement of thigh muscle HU value may become a valuable tool in etiologic studies of hip fracture and may serve as a treatment endpoint for therapies that reduce fracture risk and maintain performance by strengthening muscle.
